# Identification of 3′‐terminal 2′‐O‐methylated miRNA in plasma as a novel diagnostic biomarker of NSCLC

**DOI:** 10.1002/ctm2.1454

**Published:** 2023-11-23

**Authors:** Xu Guo, Hongyuan Guo, Kuan‐Chen Lu, Xin Yan, Jiehao Chen, Yanting Wei, Jiayi Han, Wu Sun, Huanhuan Hu, Yuanyuan Su, Lingyan Shen, Yujing Zhang, Kai Yin, Chen‐Yu Zhang, Zheng Fu

**Affiliations:** ^1^ Nanjing Drum Tower Hospital Center of Molecular Diagnostic and Therapy State Key Laboratory of Pharmaceutical Biotechnology, Jiangsu Engineering Research Center for MicroRNA Biology and Biotechnology, NJU Advanced Institute of Life Sciences, Institute of Artificial Intelligence Biomedicine, School of Life Sciences, Nanjing University Nanjing Jiangsu China; ^2^ Department of Vascular Surgery Nanjing Drum Tower Hospital, Affiliated Hospital of Medical School, Nanjing University Nanjing Jiangsu China; ^3^ Department of Respiratory and Critical Care Medicine Nanjing Drum Tower Hospital, The Affiliated Hospital of Nanjing University Medical School Nanjing China; ^4^ Department of Epidemiology and Biostatistics School of Public Health, Nanjing Medical University Nanjing Jiangsu China; ^5^ The First Department of Breast Cancer Tianjin Medical University Cancer Institute and Hospital, National Clinical Research Center for Cancer Tianjin China; ^6^ Department of Oncology Nanjing Drum Tower Hospital, The Affiliated Hospital of Nanjing University Medical School Nanjing Jiangsu China; ^7^ Institute of Artificial Intelligence Biomedicine Nanjing University Nanjing Jiangsu China; ^8^ Department of General Surgery Taixing Hospital Affiliated to Yangzhou University Taixing Jiangsu China; ^9^ Research Unit of Extracellular RNA Chinese Academy of Medical Sciences Nanjing Jiangsu China; ^10^ Pingshan Translational Medicine Center Shenzhen Bay Laboratory Shenzhen Guangdong China

Dear Editor,

Since the recent discovery of 3′‐terminal 2′‐O‐methylated (3′t‐2′Ome) miRNAs in mammals, their presence has been reported in tumour tissues and a significant difference in the methylation level has been observed between tumour and normal tissues.^1^, ^2^ However, the clinical relevance of 3′t‐2′Ome miRNAs in the circulation of cancer patients remained unknown. In this study, we speculated that the 3′t‐2′Ome miRNAs can be secreted from tumour cells into circulation, which may reflect the formation and progression of tumours.^3^, ^4^ Herein, we thoroughly investigated the 3′t‐2′Ome miRNA profile in the plasma of non‐small cell lung cancer (NSCLC) patients to evaluate their diagnostic value.

We designed a multi‐stage case–control study with a total of 149 NSCLC patients and 146 control subjects enrolled to identify 3′t‐2′Ome miRNAs in plasma as potential biomarkers for NSCLC diagnosis (Figure 1). The statistical information and clinical features of the patients are detailed in Table 1. The analysis revealed no noticeable differences in the distribution of smoking (*p* = .152), age (*p* = .773), gender (*p* = .594) or family history of cancer (*p* = .106) between the cancer‐afflicted patients and the control subjects, but there was a significant disparity in other underlying diseases (*p* < .001).

**FIGURE 1:**
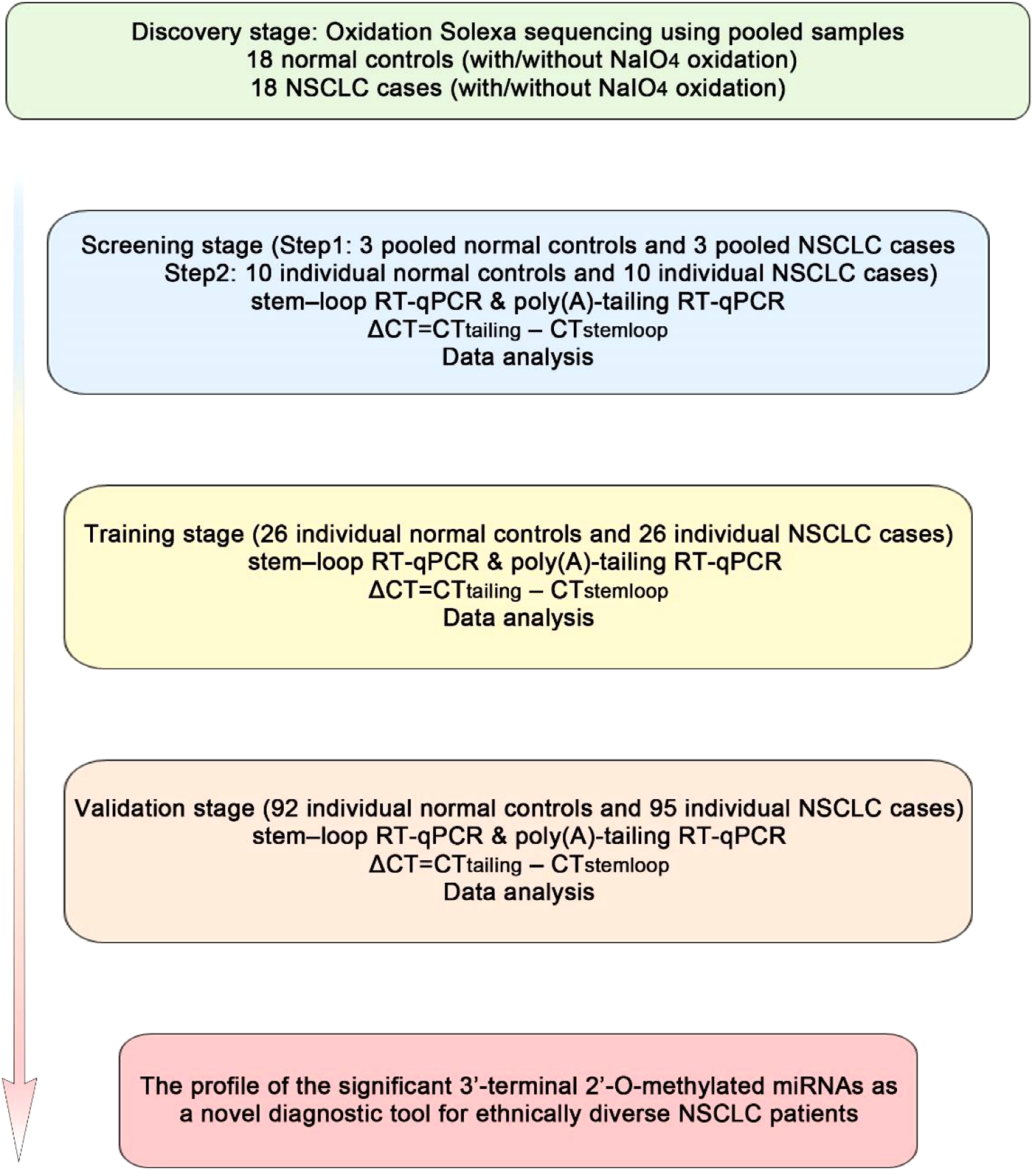
Overall study design and number of non‐small cell lung cancer (NSCLC) patients and healthy controls (including screening stage, training stage and validation stage).

**TABLE 1:** Demographic and clinical features of non‐small cell lung cancer (NSCLC) patients and control subjects in the study.

	Discovery stage		Screening stage		Training stage		Validation stage	
	Normal (*n* = 18)	NSCLC (*n* = 18)	Normal (*n* = 10)	NSCLC (*n* = 10)	Normal (*n* = 26)	NSCLC (*n* = 26)	Normal (*n* = 92)	NSCLC (*n* = 95)
Age (years) (*p* = .773)								
Mean (SD)	46.4 (19.6)	59.0 (7.2)	50.9 (14.7)	61.3 (13.4)	48.9 (15.5)	57.0 (11.8)	52.8 (16.3)	58.9 (9.6)
<60	10 (55.6%)	11 (61.1%)	5 (50.0%)	5 (50.0%)	13 (50.0%)	14 (53.8%)	45 (48.9%)	47 (49.5%)
≥0	8 (44.4%)	7 (38.9%)	5 (50.0%)	5 (50.0%)	13 (50.0%)	12 (46.2%)	47 (51.1%)	48 (50.5%)
Tumor stage								
I	–	8 (44.4%)	–	5 (50.0%)	–	10 (38.5%)	–	32 (33.7%)
II	–	5 (27.8%)	–	3 (30.0%)	–	8 (30.8%)	–	40 (42.1%)
III	–	4 (22.2%)	–	2 (20.0%)	–	7 (26.9%)	–	23 (24.2%)
IV	–	1 (5.6%)	–	0 (0%)	–	1 (3.8%)	–	0 (0%)
Sex (*p* = .594)								
Male	11 (61.1%)	9 (50.0%)	6 (60.0%)	5 (50.0%)	15 (57.7%)	16 (61.5%)	47 (51.1%)	46 (48.4%)
Female	7 (38.9%)	9 (50.0%)	4 (40.0%)	5 (50.0%)	11 (42.3%)	10 (38.5%)	45 (48.9%)	49 (51.6%)
Smoking status (*p* = .152)								
Ever and current	3 (16.7%)	3 (16.7%)	2 (20.0%)	4 (40.0%)	5 (19.2%)	7 (26.9%)	18 (19.6%)	25 (26.3%)
Never	15 (83.3%)	15 (83.3%)	8 (80.0%)	6 (60.0%)	21 (80.8%)	19 (73.1%)	74 (80.4%)	70 (73.7%)
Family history of cancer (*p* = .106)								
No	17 (94.4%)	16 (8.9%)	10 (100.0%)	10 (100.0%)	26 (100.0%)	25 (96.2%)	87 (94.6%)	85 (89.5%)
Yes	1 (5.6%)	2 (11.1%)	0 (.0%)	0 (.0%)	0 (.0%)	1 (3.8%)	5 (5.4%)	10 (10.5%)
Other basic diseases (*p* < .001)								
No	18 (100.0%)	15 (83.3%)	10 (100.0%)	8 (80.0%)	26 (100.0%)	20 (76.9%)	92 (100.0%)	66 (69.5%)
Yes	0 (.0%)	3 (16.7%)	0 (.0%)	2 (20.0%)	0 (.0%)	6 (23.1%)	0 (.0%)	29 (30.5%)

*Note*: Data are *n* (%), unless noted otherwise. *p*‐Value of two‐sided chi‐squared test.

Abbreviation: SD, standard deviation.

Deep sequencing technology allows for comprehensive profiling of small RNA expression across the entire genome, making it an ideal tool for studying small RNA species. However, 3′t‐2′Ome modification of small RNAs can reduce the efficiency of adaptor ligation, resulting in a bias against these modified small RNAs in sequencing.^2^, ^5^, ^6^ To address this issue, oxidising agent sodium periodate was used to selectively degrade and convert the unmodified small RNAs while leaving the 3′t‐2′Ome RNAs intact. This allows for a more accurate detection and quantification of the 3′t‐2′Ome RNAs compared to the unmodified ones in the sequencing library.

In line with our expectations, our findings indicate that most mammalian miRNAs may not exhibit 3′t‐2′Ome modification. Nevertheless, several miRNAs isolated from NSCLC patients demonstrated resistance to oxidation. A heatmap revealed distinct profiles of 3′t‐2′Ome miRNAs between NSCLC patients and control subjects (Figure 2A). The majority of miRNAs in control subjects had an unmodified 3′‐terminal 2′‐OH, making them susceptible to oxidation, and the copy of miRNAs significantly decreased after incubated with sodium periodate. Conversely, a stable detection of methylated miRNAs in NSCLC patients with antioxidant properties can be accomplished via deep sequencing (Figure 2B). On the other hand, piRNAs showed no significant decrease in read counts after oxidation, which also provides initial validation of the perchlorate oxidation process. Additionally, qPCR analysis determining the oxidation status of methylated and unmethylated miRNA standard samples further confirmed the oxidising effect of perchlorate on unmethylated miRNAs. Indeed, NSCLC samples have demonstrated more resistance to oxidants, and 22 methylated miRNA candidates were identified and included for further validation (Figure 2C).

**FIGURE 2:**
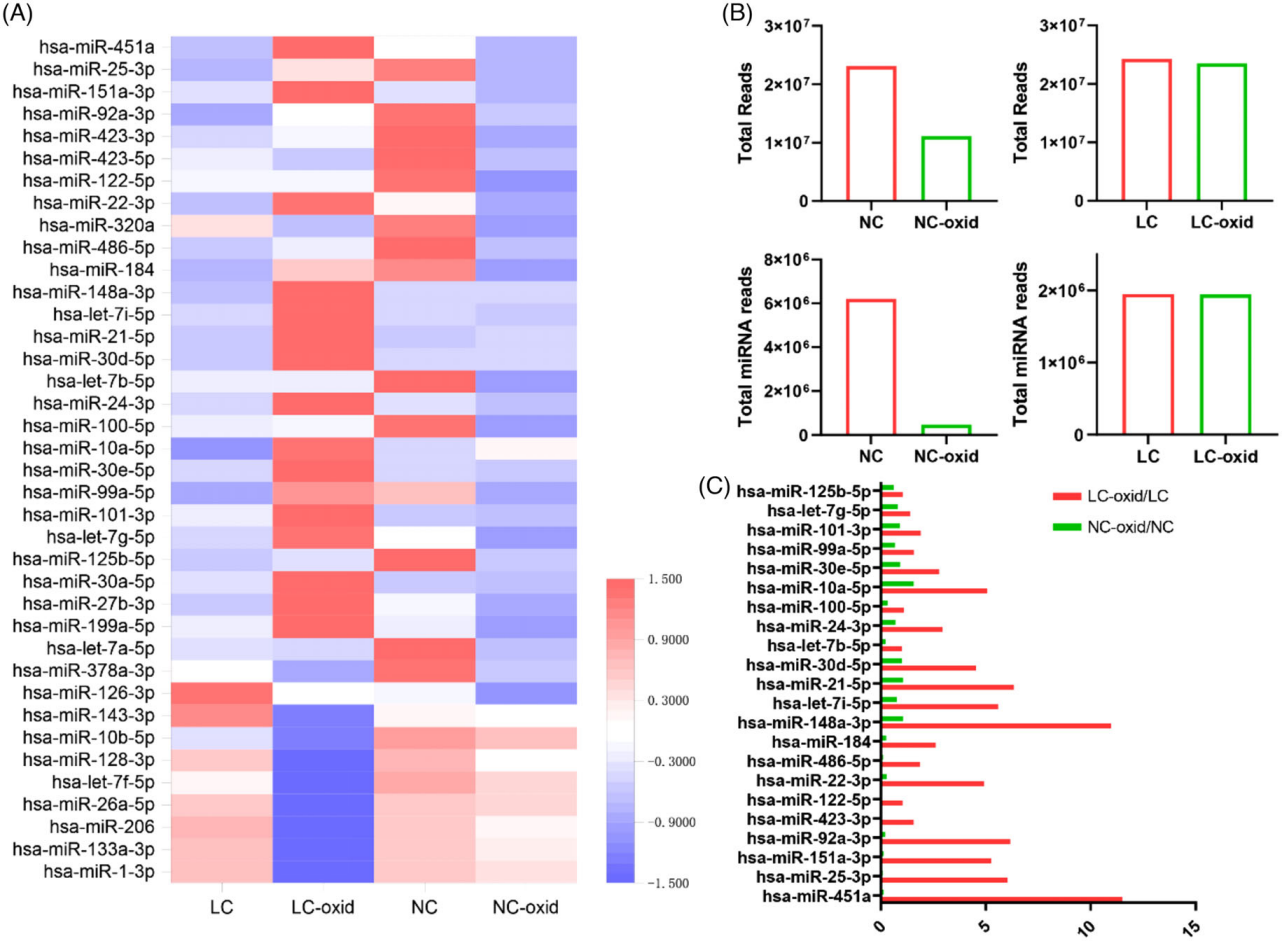
Detection of the 3′‐terminal 2′‐O‐methylated (3′t‐2′Ome) pattern of miRNA in plasma of non‐small cell lung cancer (NSCLC) patients and control subjects. (A) Heatmap of 2′Ome at the 3′‐end of human miRNAs in plasma of control subjects (NC) and NSCLC patients (LC) after oxidation procedure. NC‐oxid: control subjects with oxidation; LC‐oxid: NSCLC patients with oxidation. (B) Reads of the plasma small RNAs in oxidised and unoxidised groups were compared. The absolute reads of small RNAs and miRNAs are indicated. (C) Twenty‐two miRNAs with significantly elevated levels of 3′t‐2′Ome in plasma in NSCLC patients compared with control subjects.

Furthermore, we conducted initial screening of 22 miRNAs using mixed plasma samples from NSCLC patients and control subjects by stem‐loop reverse transcript quantitative PCR (RT‐qPCR) and poly(A)‐tailing RT‐qPCR as previously described.^7^, ^8^ The 3′t‐2′Ome levels of nine miRNAs were significantly elevated in NSCLC samples (Table S1). Subsequently, 3′t‐2′Ome levels of these nine miRNAs were measured in 10 NSCLC patients and 10 control subjects. The methylation levels of six 3′t‐2′Ome miRNAs were validated to be upregulated, including miR‐21, miR‐25, miR‐148a, miR‐122, Let‐7g and Let‐7i (Figure S3). These miRNAs were then measured in a training cohort consisting of 26 NSCLC patients and 26 control subjects, and the methylation levels of all six miRNAs were shown to be significant as shown in Figure S4 and Table S2.

Next, in a validation stage including 95 plasma samples of NSCLC patients and 92 control subjects, three 3′t‐2′Ome miRNAs, miR‐122, Let‐7g and Let‐7i, of the six miRNAs screened by training stage were significantly increased in NSCLC patients compared to control subjects (Figure 3A–F). Receiver operating characteristic curve analysis for the validation stage between NSCLC patients and control subjects is presented in Figure 3G–I. The area under the curve (AUC) of the methylation level of miR‐122, Let‐7g and Let‐7i was .9320, which performance was significantly better than the AUC of the unmethylated miRNAs (.7071) (Figures 3J and S5). Together, these findings suggest that these 3′t‐2′Ome miRNAs may originate from tumour secretion, and could serve as new biomarkers for early detection of NSCLC. In addition, we also detected the expression level of miR‐16, which was commonly used as an internal reference in miRNA biomarker studies.^9^, ^10^ Our results showed that the level of miR‐16 was not stable across the samples of different patients with similar procedure of preparation and extraction. Since the methylation level of miRNA could be detected independently of the internal control, the interference introduced by internal control could be avoided by using 3′t‐2′Ome miRNAs as a diagnostic index.

**FIGURE 3:**
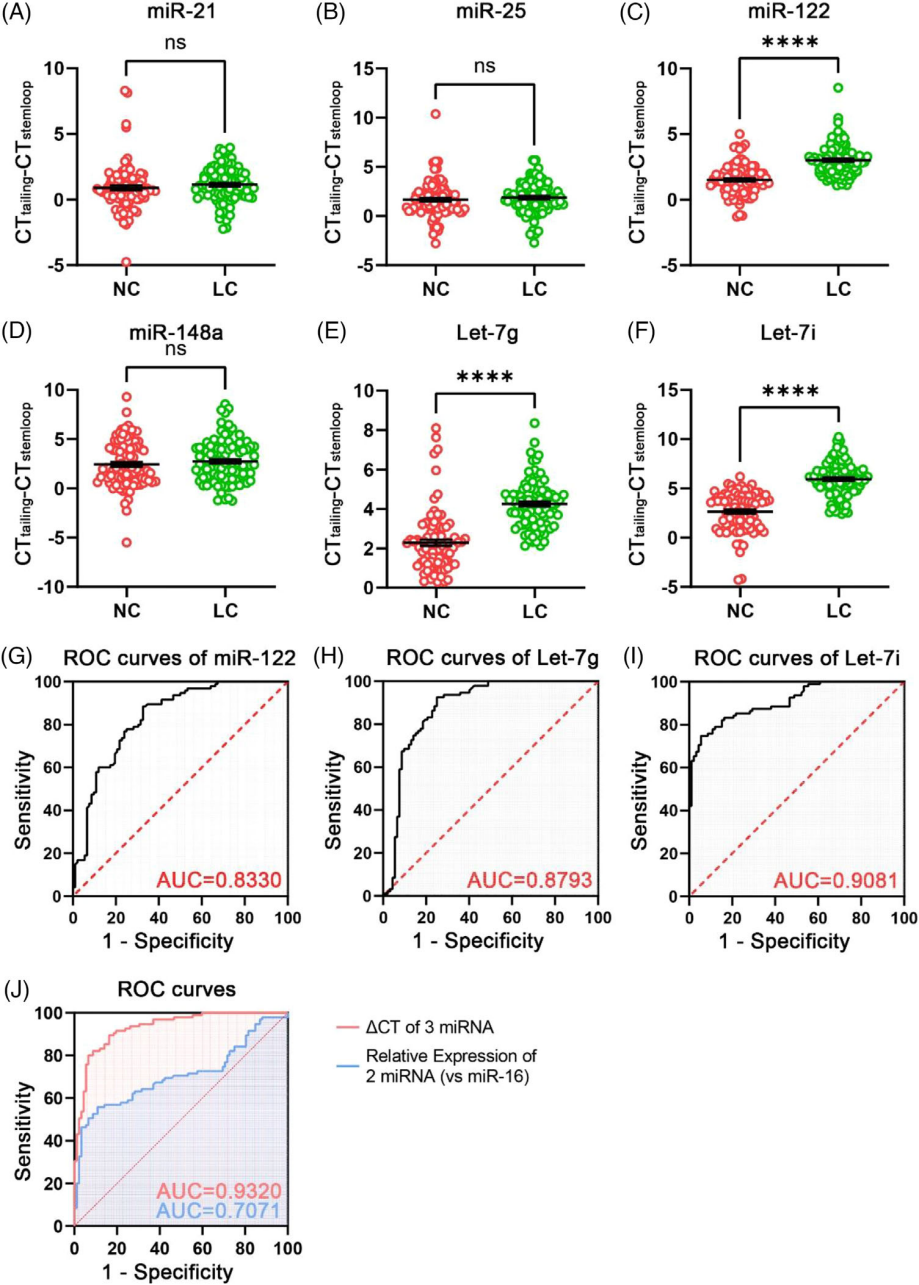
Evaluation of a panel of three 3′‐terminal 2′‐O‐methylated (3′t‐2′Ome) miRNAs as biomarkers for diagnosis of non‐small cell lung cancer (NSCLC). (A–F) Detection of the 3′t‐2′Ome pattern of six miRNAs by stem‐loop RT‐qPCR and poly(A)‐tailing RT‐qPCR in 95 plasma samples of NSCLC patients and 92 control subjects. (G–I) Receiver operating characteristic (ROC) curves for the three plasma 3′t‐2′Ome miRNA profile to distinguish NSCLC plasma samples from healthy control plasma samples in the validation set. (J) ROC curves of the three 3′t‐2′Ome miRNA panels and two miRNA panels. ^⁎⁎⁎⁎^ *p* < .0001; ns: no significant difference. Data are analysed by unpaired *t*‐test.

We also investigated the secretion ability of methylated miRNAs in vitro. When compared with normal lung epithelial cell lines HBE (healthy bronchial epithelial cells), the methylation levels of Let‐7g and Let‐7i were not only significantly higher in cells but also in exosomes harvested from the human lung cancer cell line H358 (Figure S6). Furthermore, through the utilisation of siRNA to downregulate HENMT1 expression in H358 cells, we observed a significant reduction in the methylation levels of Let‐7g and Let‐7i in the exosomes of H358 cells (Figure S6). In accordance with these results, the methylation levels of Let‐7g and Let‐7i in tumour tissues were also significantly increased in comparison with normal adjacent tissues (Figure S7).

Bioinformatic analyses have suggested that miR‐122, Let‐7g and Let‐7i might exert stimulative effects on the proliferation and migration of cancerous cells while inhibiting the process of apoptosis in NSCLC. Subsequent experimental investigations have substantiated these predictions, demonstrating that indeed miR‐122, Let‐7g and Let‐7i restrain apoptosis in H358 cells, consequently heightening their capacity for proliferation and migration. Furthermore, the study has uncovered that methylated miRNAs elicit more pronounced functional impacts than their unmethylated counterparts (Figure S8).

In conclusion, we identified a unique set of three plasma 3′t‐2′Ome miRNAs for the diagnosis of NSCLC. This is the first study to detect 3′t‐2′Ome miRNAs in human plasma and apply them to the early diagnosis of NSCLC. We anticipate that this research will lay the foundation for future investigations into the clinical value of plasma 3′t‐2′Ome miRNAs in predicting treatment efficacy, facilitating ongoing monitoring and forecasting prognosis for a broader range of tumours. However, the methods for detection of miRNA methylation used in this study were still complicated and required at least two qRT‐PCR detections for each sample. Further study would be needed for more convenient and accurate quantification of 3′t‐2′Ome miRNAs in circulation. Besides, our study only proposed the biological functions of these methylated miRNAs in vitro. Their pathological roles in disease formation are also important issues that need to be further studied in vivo.

## AUTHOR CONTRIBUTIONS

Zheng Fu, Chen‐Yu Zhang, Kai Yin and Yujing Zhang designed this study. Xu Guo, Hongyuan Guo, Kuan‐Chen Lu, Xin Yan, Jiehao Chen, Yanting Wei, Jiayi Han, Wu Sun, Huanhuan Hu, Yuanyuan Su and Lingyan Shen conducted experiments. Xu Guo, Hongyuan Guo, Kuan‐Chen Lu and Xin Yan contributed to the majority of the study. Xu Guo and Zheng Fu analysed the data. The manuscript was written by Zheng Fu, Xu Guo, and Chen‐Yu Zhang, with all authors reading and approving the final version.

## CONFLICT OF INTEREST STATEMENT

The authors declare that they have no conflicts of interest.

## DECLARATIONS

The studies involving human participants were approved by Nanjing Drum Tower Hospital (Approval number: 2019‐005‐01).

## Supporting information

Supporting information
