# Supplementary material for: Identification of 3′‐terminal 2′‐O‐methylated miRNA in plasma as a novel diagnostic biomarker of NSCLC
Source: Clin Transl Med. 2023 Nov 23;13(11):e1454. doi: 10.1002/ctm2.1454 (PMC10667623; doi:10.1002/ctm2.1454)
Supplement: Supplementary file 1 — Supporting information [file CTM2-13-e1454-s001.docx]

**Supplemental materials**

Materials and methods

Figure S1, S2, S3, S4, S5, S6, S7, S8

Table S1, S2, S3

**Materials and methods**

**Study design, patients and control subjects**

A multi-stage case-control study was designed to identify plasma 3′t-2′Ome miRNAs as a new biomarker for detecting NSCLC (Figure 1). All samples were obtained from consenting participants in accordance with the protocols approved by the ethics committees of each involved institution. Our study enrolled a total of 149 NSCLC patients and 146 control subjects. All samples were collected from Nanjing Drum Tower Hospital. During the initial biomarker discovery stage, we pooled plasma samples from 18 NSCLC patients and alongside 18 normal samples. All miRNA library construction and deep sequencing were performed by BGI. Briefly, miRNA libraries were constructed and validated according to the TruSeq Small RNA Sample Pre Kit (Illumina). Raw data for each sRNA library were generated on the Illumina HiSeq 4000 platform. The clean reads were obtained after data filtration. miRNA sequences were obtained from miRBase v21 and Bowtie was used to align clean reads to these reference sequences for annotation. Subsequently, sequential validation was performed using stem-loop RT-qPCR and poly(A)-tailing RT-qPCR to refine the plasma 2′-O-methylated miRNA signature for NSCLC detection. Initially, the sequencing stage plasma samples were mixed into three groups of equal proportion and subjected to RT-qPCR validation. Subsequently, we conducted preliminary screening using 10 NSCLC patient plasma samples and 10 normal samples. During the biomarker selection stage, we utilized a training stage comprising 26 NSCLC plasma samples and 26 control samples. Additionally, a validation stage was formed with 95 NSCLC plasma samples and 92 healthy control subjects. All patients included in this study were diagnosed with NSCLC between 2012 and 2018, and blood samples were collected prior to any therapeutic interventions, such as surgery, chemotherapy, or radiotherapy. The histopathology of patients was confirmed through surgical resection of the tumors, and tumor staging was determined based on the findings from surgery. For patients deemed unsuitable for surgical treatment, histopathology and tumor staging were confirmed via histobiopsy and imaging technology. Tumor staging adhered to the tumor-node-metastasis (TNM) staging system established by the International Union against Cancer. Histological grading was assessed according to the World Health Organization (WHO) criteria.

The demographic and clinical features of the patients are detailed in Table 1. Healthy control subjects were recruited from a sizable population undergoing regular health examinations at Nanjing Drum Tower Hospital. Control subjects were selected based on the absence of any evidence of disease and matched to patients in terms of age, gender, and ethnicity.

**Plasma preparation, RNA isolation**

Venous blood samples (~ 5 ml) were collected from each patient and healthy volunteer. In the case of NSCLC patients, blood samples were obtained prior to any therapeutic interventions and before surgical resections of the primary tumors. RNA was isolated using the miRcute miRNA isolation kit, following the manufacturer's instructions (TIANGEN). A comprehensive description of the experimental protocols as follows: Pipette 200μL of plasma and add it to 900μL of MZA lysis buffer, vortex for 30 seconds until completely homogenized. Incubate at room temperature for 5 minutes, then add 200μL of chloroform, and vortex vigorously for 15 seconds. Incubate at room temperature for 5 minutes. Centrifuge at 12,000 rpm and 4°C for 15 minutes. After centrifugation, the sample will separate into three layers: a yellow organic phase, a white interphase, and a colorless aqueous phase. Transfer the aqueous phase into a new 1.5mL Eppendorf tube. Slowly add 2 times the volume of anhydrous ethanol to the transferred solution (e.g., for 500μL of transferred solution, add 1mL of anhydrous ethanol), and gently invert to mix. Transfer the resulting solution and precipitate to the miRelute column, incubate at room temperature for 2 minutes, then centrifuge at room temperature and 12,000 rpm for 30 seconds, and discard the filtrate. Add 700μL of MRD protein precipitation solution (previously mixed with ethanol) to the miRelute column, let stand at room temperature for 2 minutes, then centrifuge at room temperature and 12,000 rpm for 30 seconds, and discard the waste liquid. Add 500μL of wash buffer RW (previously mixed with ethanol) to the miRelute column, let stand at room temperature for 2 minutes, then centrifuge at room temperature and 12,000 rpm for 30 seconds, and discard the waste liquid. Repeat the washing step once more. Centrifuge at room temperature and 12,000 rpm for 2 minutes, and discard the collection tube. Transfer the miRelute column to a new RNase-Free 1.5mL centrifuge tube, add 20μL of DEPC water to the center of the adsorption membrane, incubate at room temperature for 2 minutes, then centrifuge at room temperature and 12,000 rpm (approximately 13,400g) for 2 minutes. Store the RNA at -80°C in an ultra-low temperature freezer.

**Oxidation RNA deep sequencing**

RNAs were resuspended in 1× borate/borax buffer, either with or without the addition of sodium periodate (NaIO_4_) (final concentration: 25 mM) (Sigma-Aldrich), and incubated for 30 minutes at room temperature in the dark. Following the addition of 1/10 volume of 100% glycerol, samples were further incubated for 10 minutes at room temperature in the dark.[1] Upon purification, samples were precipitated using ethanol and employed as input for RNA deep sequencing via the Illumina 3000 platform (The Beijing Genomics Institute). Briefly, following the PAGE purification of small RNA molecules under 30 bases, a pair of adaptors were ligated to their 5′ and 3′ ends. The small RNA molecules were then amplified using adaptor primers for 17 cycles, and fragments of approximately 90 bp (small RNA + adaptors) were isolated from agarose gels. The purified DNA was used directly for cluster generation and sequencing analysis using Illumina's Sequencer, following the manufacturer's instructions. The image files generated by the sequencer were then processed to produce digital-quality data. Subsequent procedures included summarizing the generated data, evaluating sequencing quality, and calculating the length distribution of small RNA reads. Ultimately, clean reads were compared with the miRBase database (Release 22.1).

**RT-qPCR assays**

Our research employed two RT-qPCR systems: the stem-loop system and the poly(A)-tailing system. [2, 3]A TaqMan probe-based RT-qPCR assay for miRNA detection was carried out according to the manufacturer's instructions (Applied Biosystems), with a minor modification as previously described. The reverse transcription system consisted of dNTPs with dTTP (100 mM), MultiScribe Reverse Transcriptase (50 U/µL), 10X RT Buffer, RNase Inhibitor (20 U/µL), nuclease-free water, and a stem-loop RT primer (Applied Biosystems). For cDNA synthesis, reaction mixtures were incubated at 16°C for 30 minutes, at 42°C for 30 minutes, at 85°C for 5 minutes, and then held at 4°C. Real-time PCR was performed (1 cycle of 95°C for 5 minutes and 40 cycles of 95°C for 15 seconds and 60°C for 1 minute) using an Applied Biosystems LightCycler480 Detection System. The reaction was executed with a final volume of 20 µL, containing 2.5 µL of cDNA, 0.33 µL of hydrolysis probe (Applied Biosystems), 10 µL of 2× Universal Master Mix II (Applied Biosystems), and 7.17 µL of DEPC water.

The poly(A)-tailing RT-qPCR assay for miRNA detection was performed according to the manufacturer's instructions (Qiagen). The reverse transcription system included 10 × miScript Nucleics Mix, 5 × miScript HiSpec Buffer, and miScript Reverse Transcriptase Mix. Samples were kept on ice before initiating the programs. The reverse transcription program was as follows: 37°C for 60 minutes, 95°C for 5 minutes, and then held at 4°C. Upon obtaining cDNAs, 2 × QuantiTect SYBR Green Master Mix, 10 × miScript Universal Primer, and 10 × miScript Primer Assay were used for real-time PCR detection. The program was set as follows: 95°C for 15 minutes, followed by 40 cycles of 94°C for 15 seconds, 55°C for 30 seconds, and 72°C for 30 seconds. Each cDNA sample was tested in duplicate.

**Cell cultures and reagents**

The human lung cancer cell line H358 and human bronchial epithelial cell line HBE were procured from iCell Bioscience Inc (Shanghai, China). Cells were maintained in RPMI-1640 medium supplemented with 10% FBS, 100 U/mL penicillin, and 100 mg/mL streptomycin (Life Technologies/Gibco, Grand Island, New York). The cells were passaged every three days at a 1:3 ratio.

**Exosome isolation and analyses**

H358 and HBE cells were cultured in serum-free Opti-MEM for 36 h. The cell culture medium was then harvested from cells and centrifuged at 2000× g for 30 min. The supernatant containing the cell-free culture medium was transferred to a new tube without disturbing the pellet. Subsequently, total exosomes were isolated by Total Exosome Isolation (from cell culture media) (Invitrogen, 4478359) according to the manufacturer’s instruction. The exosome pellet was resuspended in PBS, Trizol Reagent or RIPA lysis buffer. The exosomes resuspended in Trizol Reagent were subjected to qRT-PCR. The exosomes resuspended in RIPA lysis buffer were subjected to western blotting for specific exosome markers CD9 (Proteintech, 20597-1-AP), CD63 (Proteintech, 25682-1-AP) and TSG101 (Proteintech, 28283-1-AP). The exosomes resuspended in PBS were subjected to nanoparticle tracking analysis.

Isolated exosomes were analyzed with Nanosight LM10, a system equipped with a blue laser (405 nm). The nanoparticle is illuminated by a laser, and its motion in Brownian motion is captured for 60 s. At least 5 videos were collected from each individual sample to provide representative concentration measurements, and all control samples were run alongside experimental samples. The size distribution curves were evaluated with NTA software and were averaged within each sample in the video repetition, and then averaged between repetitions to provide a representative size distribution.

**Cell proliferation assay**

We detected cell proliferation by CCK-8 assay, H358 cells were plated at 2 × 10^4^ cells per well in 96-well plates and incubated overnight in RPMI-1640 supplemented with 10% FBS. The cell proliferation index was measured using a Cell Counting Kit-8 (CK04-500, Dojindo, Japan) at 12, 24, 36, 48, and 60 h post-transfection according to the manufacturer’s instruction. Absorbance was measured at a wavelength of 450 nm.

**Wound healing assays**

H358 cells were seeded into 6-well plates. Then, the cell layer was gently wounded using a plastic pipette tip (P200) and rinsed with PBS before the culture medium was replaced. The bottom of the wells was marked to indicate where the initial images of the wounded area were captured. At 24 h of incubation, images (10×) of the same areas were recorded using a photomicroscope (BX51 Olympus, Japan), and closure of the wounds was processed using Image-Pro Plus 6.0

**Apoptosis assay**

The H358 cells were transfected with methylated miRNA, unmethylated miRNA or constructs for 24~36 hours at indicated concentrations and stained with Annexin V-FITC and PI (BD Biosciences, 556547). Apoptosis was analyzed by flow cytometry (FACScalibur, BD Biosciences). Both early-apoptotic (Annexin V^+^ and PI^–^) and late-apoptotic (Annexin V^+^ and PI^+^) cells were included in the analyses.

**Statistical analysis**

Data analysis was performed using GraphPad Prism 8. The results are presented as the means ± standard error of the mean (SEM) of at least three independent experiments. Differences were considered statistically significant at P < 0.05, as determined by two-tailed Student's t-tests. The ROC curves were constructed to evaluate the specificity and sensitivity of NSCLC prediction for each miRNA individually and for the combination of miRNAs. All analyses above were performed with the use of SPSS statistical software (version 20.0).

**Reference**

1. Liang, H., et al., *3'-Terminal 2'-O-methylation of lung cancer miR-21-5p enhances its stability and association with Argonaute 2.* Nucleic Acids Res, 2020. **48**(13): p. 7027-7040.

2. Wang, N., et al., *Direct quantification of 3' terminal 2'-O-methylation of small RNAs by RT-qPCR.* RNA, 2018. **24**(11): p. 1520-1529.

3. Kong, Y., et al., *Accurate quantification of 3'-terminal 2'-O-methylated small RNAs by utilizing oxidative deep sequencing and stem-loop RT-qPCR.* Front Med, 2022. **16**(2): p. 240-250.


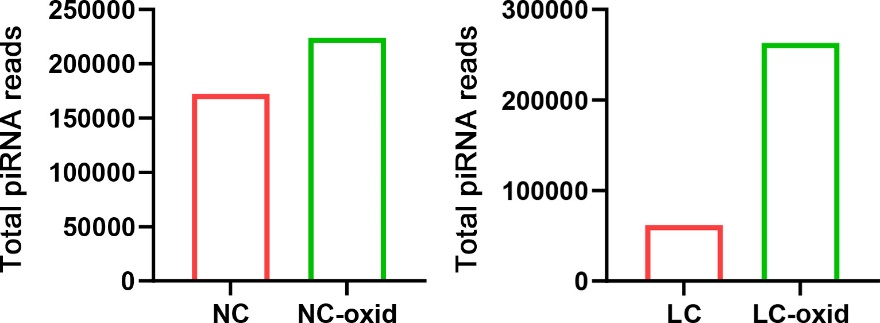
Figure S1 Reads of the piRNAs in oxidized and unoxidized plasma RNA samples of NSCLC patients and control subjects.


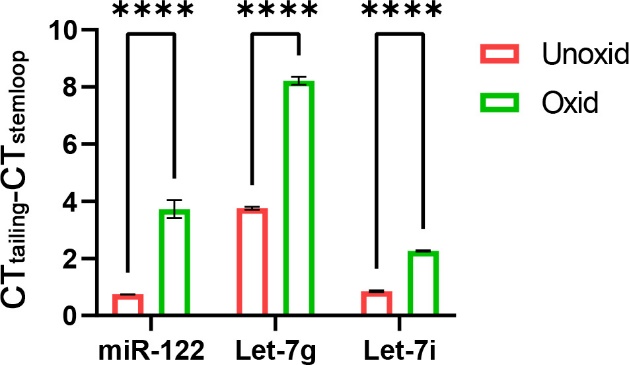
Figure S2 Detection of the 3′-terminal 2′-O-methylation level of three miRNA standard samples (The mixture consisted of miRNA standards and methylated miRNA standards in a 1:1 ratio) by stem–loop RT-qPCR and poly(A)-tailing RT-qPCR with/without oxidation. Oxid: miRNA standard samples with oxidation. Unoxid: miRNA standard samples without oxidation. ⁎⁎⁎⁎: P < 0.0001. Data are analysed by 2-way ANOVA.


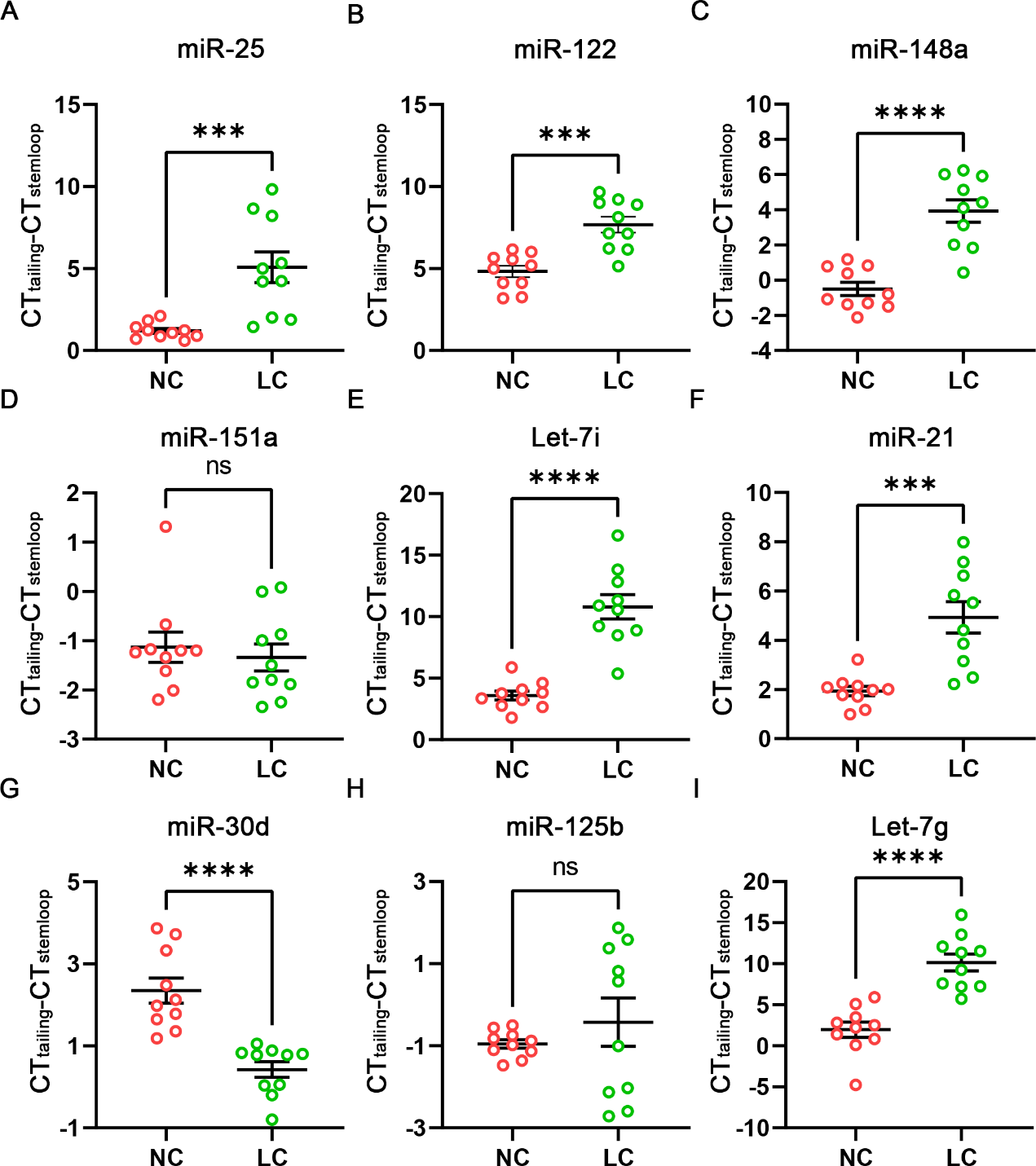


Figure S3 Detection of the 3′-terminal 2′-O-methylation pattern of 9 miRNAs by stem–loop RT-qPCR and poly(A)-tailing RT-qPCR in 10 NSCLC patients and control subjects. The asterisks indicate significant differences from control subjects. ⁎⁎⁎: P < 0.001; ⁎⁎⁎⁎: P < 0.0001; ns: no significant difference. Data are analysed by unpaired t test.


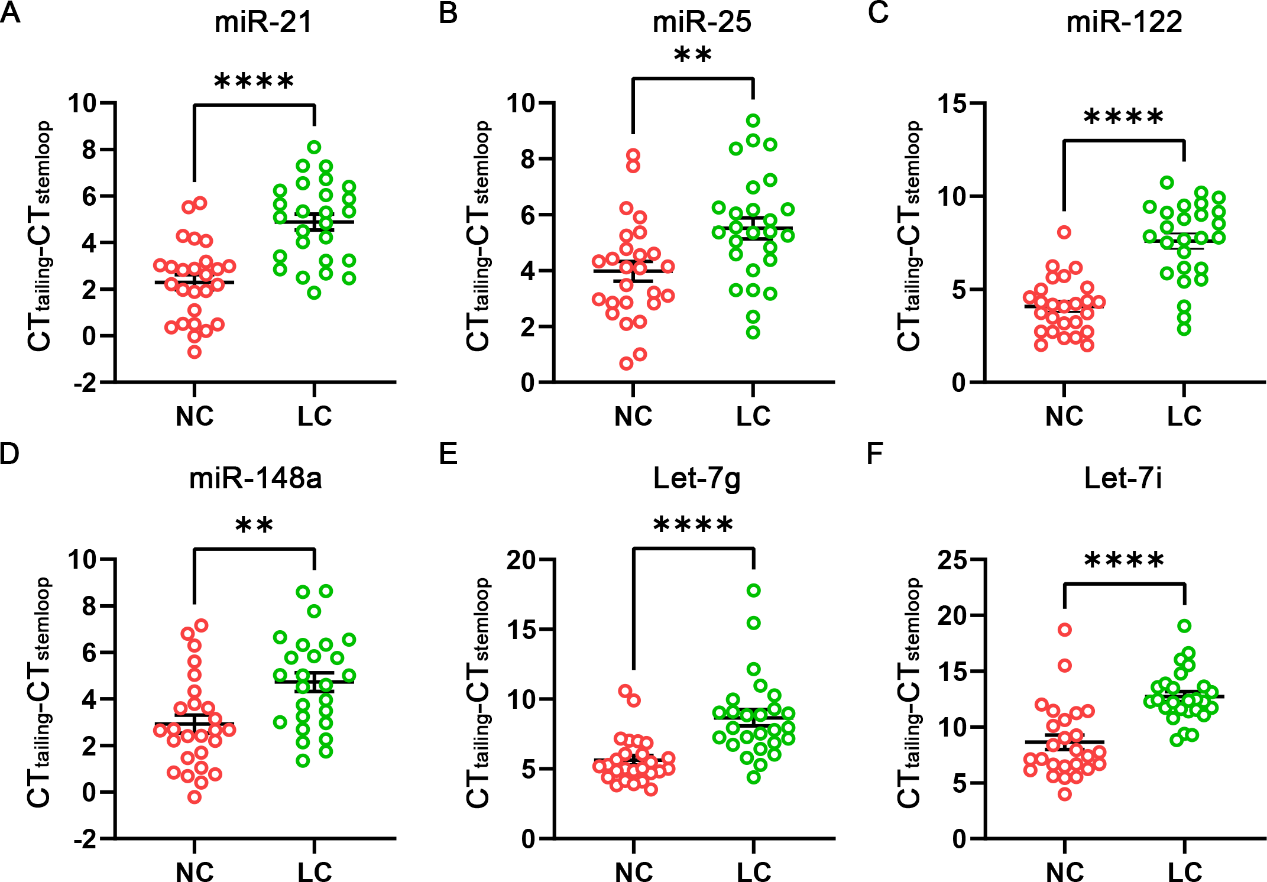


Figure S4 Detection of the 3′-terminal 2′-O-methylation pattern of 6 miRNAs by stem–loop RT-qPCR and poly(A)-tailing RT-qPCR in 26 NSCLC patients and control subjects. The asterisks indicate significant differences from control subjects. ⁎⁎: P < 0.01; ⁎⁎⁎⁎: P < 0.0001. Data are analysed by unpaired t test.


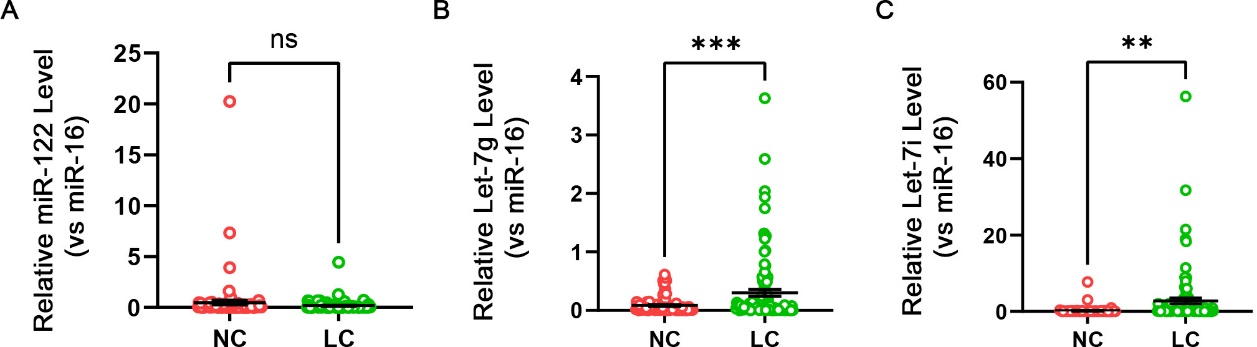


Figure S5 Quantified the relative contents of the selected three miRNAs in the plasma from patient with NSCLC in the validation set by stem–loop RT-qPCR. ⁎⁎: P < 0.01; ⁎⁎⁎: P < 0.001; ns: no significant difference. Data are analysed by unpaired t test.


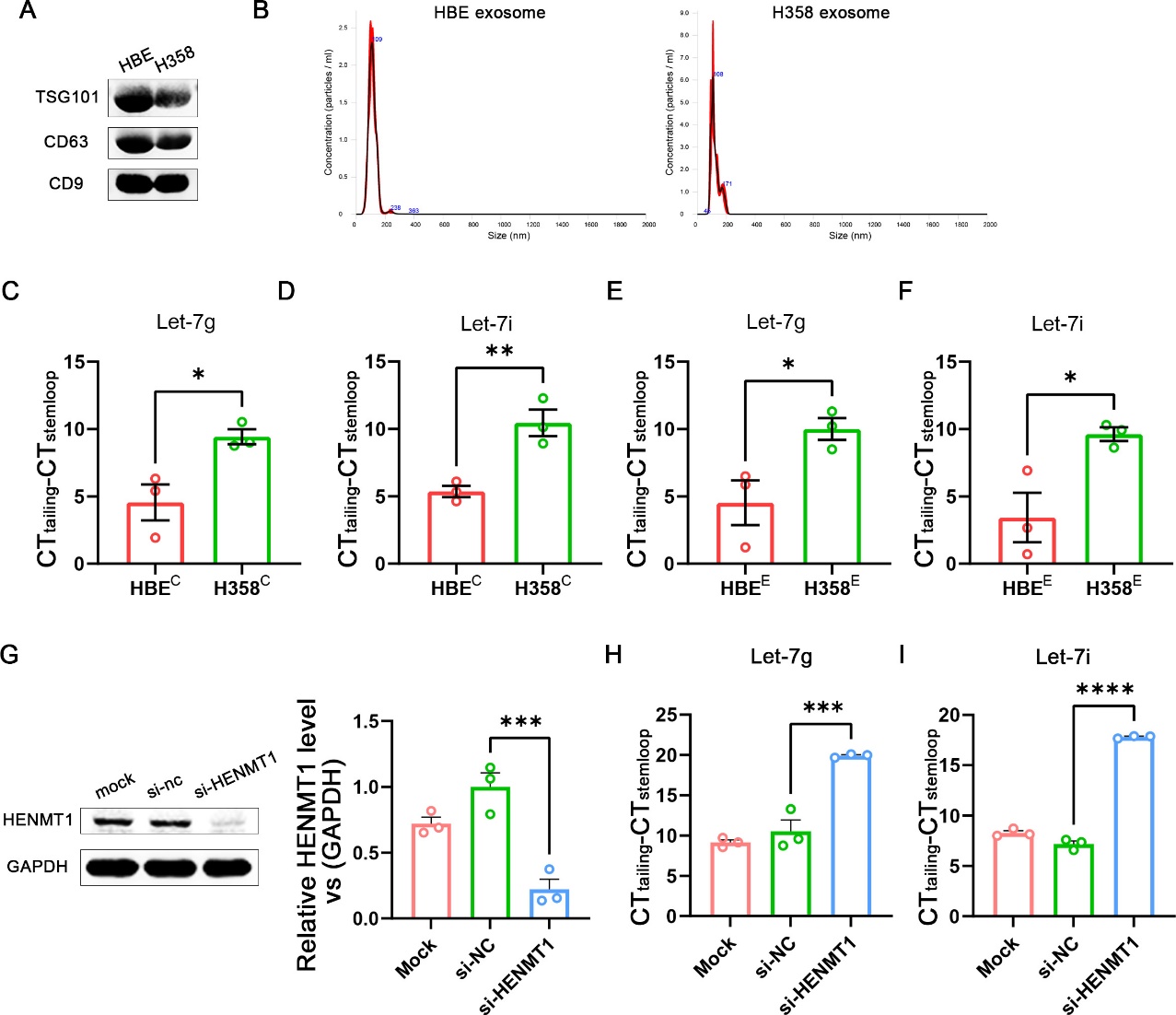


Figure S6 Detection of the 3′-terminal 2′-O-methylation pattern of the selected three miRNAs in cell and exosome. (A) Western blot analysis of the exosomes markers CD9, CD63 and TSG101. (B) Nanoparticle tracking analysis (NTA) of exosomes isolated from the conditioned medium of HBE and H358. (C, D) Detection of the 3′-terminal 2′-O-methylation pattern of the selected three miRNAs by stem–loop RT-qPCR and poly(A)-tailing RT-qPCR in HBE cell and H358 cell. (E, F) Detection of the 3′-terminal 2′-O-methylation pattern of the selected three miRNAs by stem–loop RT-qPCR and poly(A)-tailing RT-qPCR in HBE exosome and H358 exosome. (G) Western blot analysis of HENMT1 in H358. (H, I) Detection of the 3′-terminal 2′-O-methylation pattern of the selected three miRNAs by stem–loop RT-qPCR and poly(A)-tailing RT-qPCR after transfection. ⁎: P < 0.05; ⁎⁎: P < 0.01; ⁎⁎⁎: P < 0.001; ⁎⁎⁎⁎: P < 0.0001. Data are analysed by unpaired t test (C, D, E, F) and 1-way ANOVA (G, H, I).


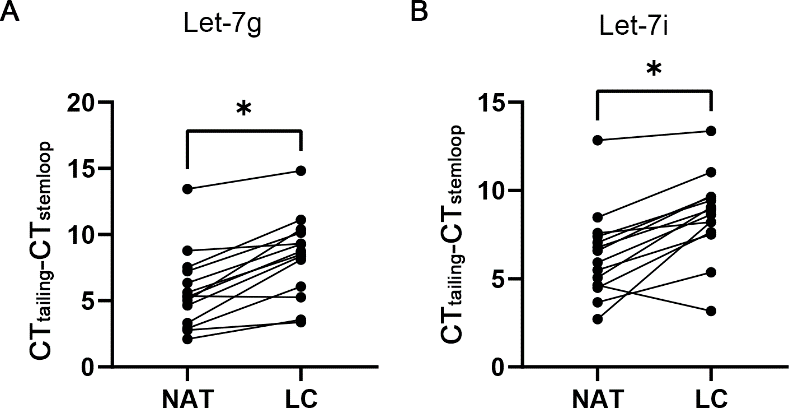


Figure S7 Detection of the 3′-terminal 2′-O-methylation pattern of the selected three miRNAs by stem–loop RT-qPCR and poly(A)-tailing RT-qPCR in NSCLC tumor tissues (LC) and normal adjacent tissues (NAT). ⁎: P < 0.05. Data are analysed by unpaired t test.


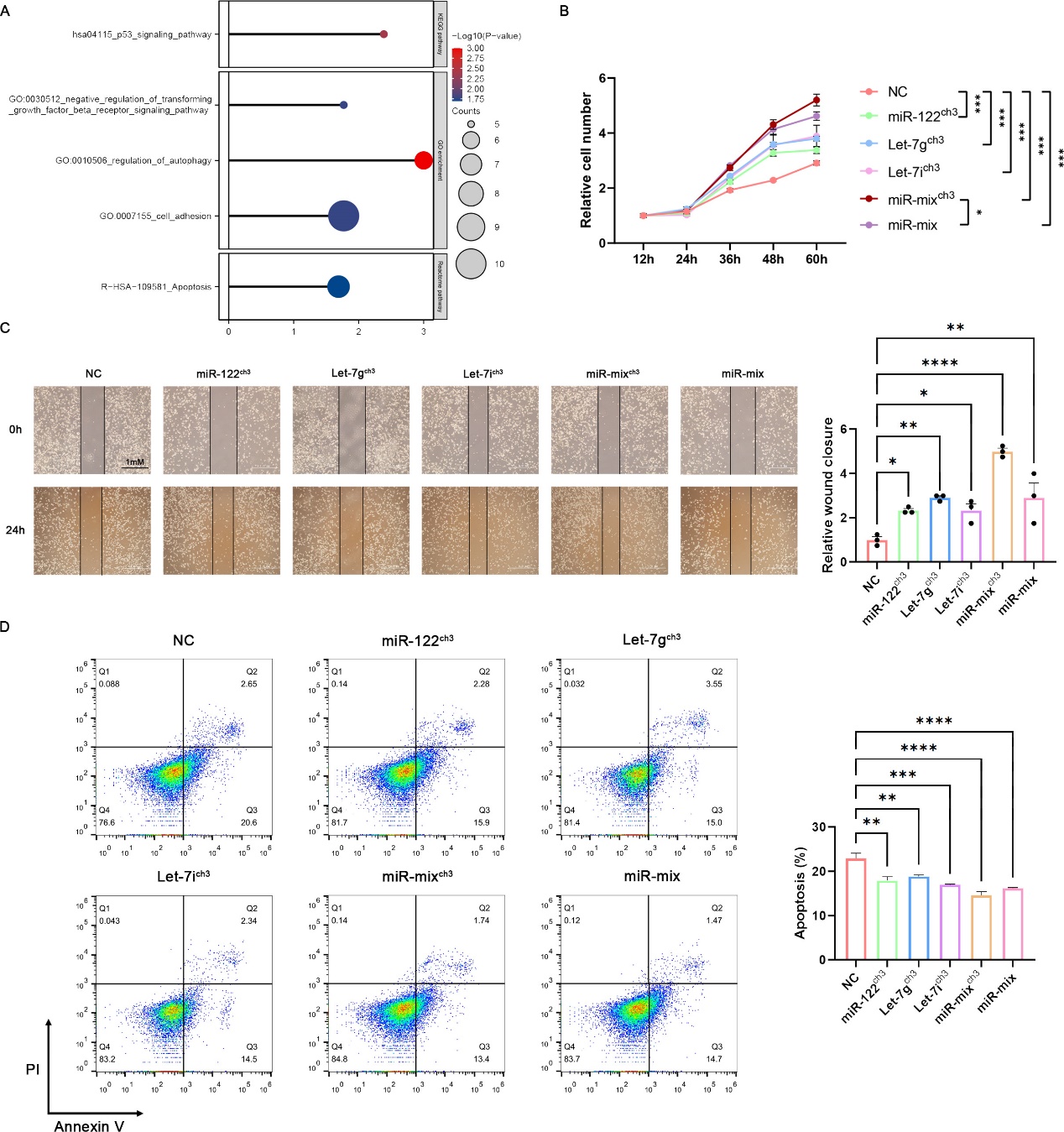
Figure S8 3′-terminal 2′-O-methylated miR-122, Let-7g and Let-7i promote proliferation and migration while inhibiting apoptosis in H358 cells. (A) Bioinformatics analysis of the potential functions of miR-122, Let-7g and Let-7i in NSCLC. (B) Cell proliferation assays (CCK-8) were performed 12, 24, 36, 48 and 60 h after the transfection of H358 cells. (C) Scratch assay showing the migration of H358 cells (n = 3). (D) Apoptosis assays were performed 24 h after the transfection of H358 cells. ⁎: P < 0.05; ⁎⁎: P < 0.01, ⁎⁎⁎: P < 0.001; ⁎⁎⁎⁎: P < 0.0001. Data are analysed by 2-way ANOVA (B) and 1-way ANOVA (C, D).

Table S1 CT value differences between stem-loop RT-qPCR and poly(A)-tailing RT-qPCR of 22 miRNAs from mixed plasma samples

|  | NC | | | LC | | |  |
| --- | --- | --- | --- | --- | --- | --- | --- |
|  | CT_Tailing_ | CT_Stemloop_ | ΔCT | CT_Tailing_ | CT_Stemloop_ | ΔCT | P value |
| hsa-miR-451a | 32.04  (0.49) | 27.29  (0.60) | 4.75  (0.14) | 33.79  (0.93) | 28.02  (0.12) | 5.77  (0.83) | 0.159 |
| hsa-miR-148a-3p | 32.85  (0.13) | 27.61  (0.48) | 5.24  (0.50) | 36.54  (1.88) | 27.60  (0.53) | 8.940  (1.35) | 0.022 |
| hsa-miR-21-5p | 26.01  (0.77) | 25.88  (0.66) | 0.13  (0.45) | 33.63  (1.09) | 27.45  (0.18) | 6.18  (1.10) | 0.002 |
| hsa-miR-92a-3p | 32.59  (0.61) | UD | - | 32.82  (0.15) | UD | - | - |
| hsa-miR-25-3p | 29.46  (0.45) | 27.73  (0.56) | 1.73  (0.49) | 34.47  (0.58) | 27.31  (0.89) | 7.16  (0.63) | <0.001 |
| hsa-let-7i-5p | 26.84  (0.54) | 25.63  (0.86) | 1.21  (0.38) | 32.18  (0.30) | 23.90  (0.33) | 8.29  (0.26) | <0.001 |
| hsa-miR-151a-3p | 26.44  (1.20) | 23.52  (2.39) | 2.92  (1.19) | 29.03  (0.55) | 23.11  (0.43) | 5.93  (0.47) | 0.03 |
| hsa-miR-10a-5p | 31.91  (0.42) | 34.47  (0.75) | -2.56  (0.33) | 32.72  (0.15) | 34.94  (1.83) | -2.22  (1.68) | 0.795 |
| hsa-miR-22-3p | 25.26  (1.10) | 26.46  (0.76) | -1.20  (0.51) | 27.50  (0.18) | 27.35  (0.32) | 0.16  (0.49) | 0.053 |
| hsa-miR-30d-5p | 32.01  (0.18) | 29.40  (0.06) | 2.62  (0.77) | 31.00  (0.54) | 26.25  (0.72) | 4.75  (0.18) | 0.019 |
| hsa-miR-24-3p | 26.17  (0.95) | 35.52  (0.78) | -9.35  (0.47) | 28.09  (0.09) | UD | - | - |
| hsa-miR-30e-5p | UD | UD | - | UD | UD | - | - |
| hsa-miR-184 | UD | UD | - | 38.13  (1.49) | UD | - | - |
| hsa-miR-101-3p | 32.69  (0.11) | 30.96  (2.51) | 1.73  (2.40) | 32.15  (0.57) | 28.12  (0.61) | 4.04  (0.11) | 0.246 |
| hsa-miR-486-5p | 26.63  (0.06) | 26.87  (1.12) | -0.24  (1.06) | 24.54  (1.01) | 26.07  (0.43) | -1.53  (1.07) | 0.29 |
| hsa-miR-99a-5p | 27.15  (0.52) | 28.29  (0.09) | -1.14  (0.47) | 25.25  (1.13) | 28.41  (0.87) | -3.17  (0.94) | >0.999 |
| hsa-miR-423-3p | UD | UD | - | UD | UD | - | - |
| hsa-let-7g-5p | 29.53  (0.65) | 23.22  (0.27) | 6.31  (0.60) | 34.76  (0.09) | 22.55  (0.82) | 12.21  (0.90) | 0.002 |
| hsa-miR-100-5p | 25.82  (0.84) | 35.03  (1.13) | -9.21  (0.32) | 27.77  (0.12) | 36.89  (0.96) | -9.11  (1.04) | 0.904 |
| hsa-miR-125b-5p | 32.84  (0.18) | 32.95  (0.52) | -0.11  (0.65) | 35.68  (0.81) | 31.60  (1.03) | 4.09  (0.77) | 0.004 |
| hsa-miR-122-5p | 28.97  (2.37) | 28.36  (2.09) | 0.61  (0.30) | 30.35  (1.66) | 22.22  (0.92) | 8.14  (1.56) | 0.003 |
| hsa-let-7b-5p | 33.65  (0.56) | 30.18  (0.84) | 3.47  (0.33) | 36.71  (0.98) | 32.36  (0.49) | 4.35  (1.14) | 0.353 |

1. The data are showed as the mean (SD).
2. P value was measured by Student’s-t test.
3. UD: undetected

Table S2 CT value differences between stem-loop RT-qPCR and poly(A)-tailing RT-qPCR of 6 miRNAs in training stage

|  | Training set | | | | Validation set | | | |
| --- | --- | --- | --- | --- | --- | --- | --- | --- |
|  | Normal（26） | NSCLC（26） | Fold change | P | Normal（92） | NSCLC（95） | Fold change | P |
| miR-21 | 2.30 (1.64) | 4.88 (1.69) | 2.13 | <0.0001 | 0.89 (1.76) | 1.14 (1.33) | 1.27 | 0.2942 |
| miR-25 | 3.97 (1.76) | 5.51 (1.90) | 1.39 | 0.0046 | 1.66 (1.80) | 1.88 (1.57) | 1.13 | 0.3779 |
| miR-122 | 4.08 (1.45) | 7.59 (2.07) | 1.86 | <0.0001 | 1.50 (1.16) | 3.01 (1.21) | 2.00 | <0.0001 |
| miR-148a | 2.92 (1.95) | 4.73 (2.00) | 1.62 | 0.0021 | 2.44 (2.34) | 2.75 (2.14) | 1.13 | 0.3583 |
| Let-7g | 5.62 (1.66) | 8.66 (2.90) | 1.54 | <0.0001 | 2.29 (1.52) | 4.26 (1.22) | 1.86 | <0.0001 |
| Let-7i | 8.63 (3.26) | 12.73 (2.27) | 1.47 | <0.0001 | 2.65 (1.95) | 5.95 (1.73) | 2.25 | <0.0001 |

1. The data are showed as the mean (SD).
2. P value was measured by Student’s-t test.

Table S3 Primer list.

|  | Qiagen | | ThermoFisher | |
| --- | --- | --- | --- | --- |
|  | GeneGlobe ID | Cat. No. | Assay ID | Cat. No. |
| hsa-miR-451a | YP02119305 | 339306 | 001141 | 4427975 |
| hsa-miR-148a-3p | YP00205867 | 339306 | 000470 | 4427975 |
| hsa-miR-21-5p | YP00204230 | 339306 | 000397 | 4427975 |
| hsa-miR-92a-3p | YP00204258 | 339306 | 000431 | 4427975 |
| hsa-miR-25-3p | YP00204361 | 339306 | 000403 | 4427975 |
| hsa-let-7i-5p | YP00204394 | 339306 | 002221 | 4427975 |
| hsa-miR-151a-3p | YP00204576 | 339306 | 002254 | 4427975 |
| hsa-miR-10a-5p | YP00204778 | 339306 | 000387 | 4427975 |
| hsa-miR-22-3p | YP00204606 | 339306 | 000398 | 4427975 |
| hsa-miR-30d-5p | YP00206047 | 339306 | 000420 | 4427975 |
| hsa-miR-24-3p | YP00204260 | 339306 | 000402 | 4427975 |
| hsa-miR-30e-5p | YP00204714 | 339306 | 000422 | 4427975 |
| hsa-miR-184 | YP00204601 | 339306 | 000485 | 4427975 |
| hsa-miR-101-3p | YP00204786 | 339306 | 002253 | 4427975 |
| hsa-miR-486-5p | YP00204001 | 339306 | 001278 | 4427975 |
| hsa-miR-99a-5p | YP00204521 | 339306 | 000435 | 4427975 |
| hsa-miR-423-3p | YP00204488 | 339306 | 002626 | 4427975 |
| hsa-let-7g-5p | YP00204565 | 339306 | 002282 | 4427975 |
| hsa-miR-100-5p | YP00205689 | 339306 | 000437 | 4427975 |
| hsa-miR-125b-5p | YP00205713 | 339306 | 000449 | 4427975 |
| hsa-miR-122-5p | YP00205664 | 339306 | 002245 | 4427975 |
| hsa-let-7b-5p | YP00204750 | 339306 | 002619 | 4427975 |
